# Host‐induced gene silencing of an important pathogenicity factor *PsCPK1* in *Puccinia striiformis* f. sp. *tritici* enhances resistance of wheat to stripe rust

**DOI:** 10.1111/pbi.12829

**Published:** 2017-10-23

**Authors:** Tuo Qi, Xiaoguo Zhu, Chenlong Tan, Peng Liu, Jia Guo, Zhensheng Kang, Jun Guo

**Affiliations:** ^1^ State Key Laboratory of Crop Stress Biology for Arid Areas College of Plant Protection Northwest A&F University Yangling Shaanxi China

**Keywords:** host‐induced gene silencing, *PsCPK1*, wheat, *Puccinia striiformis* f. sp. *tritici*, virulence, RNAi

## Abstract

Rust fungi are devastating plant pathogens and cause a large economic impact on wheat production worldwide. To overcome this rapid loss of resistance in varieties, we generated stable transgenic wheat plants expressing short interfering RNAs (siRNAs) targeting potentially vital genes of *Puccinia striiformis* f. sp. *tritici* (*Pst*). Protein kinase A (PKA) has been proved to play important roles in regulating the virulence of phytopathogenic fungi. *PsCPK1*, a PKA catalytic subunit gene from *Pst*, is highly induced at the early infection stage of *Pst*. The instantaneous silencing of *PsCPK1* by *barley stripe mosaic virus* (BSMV)‐mediated host‐induced gene silencing (HIGS) results in a significant reduction in the length of infection hyphae and disease phenotype. These results indicate that *PsCPK1* is an important pathogenicity factor by regulating *Pst* growth and development. Two transgenic lines expressing the RNA interference (RNAi) construct in a normally susceptible wheat cultivar displayed high levels of stable and consistent resistance to *Pst* throughout the T_3_ to T_4_ generations. The presence of the interfering RNAs in transgenic wheat plants was confirmed by northern blotting, and these RNAs were found to efficiently down‐regulate *PsCPK1* expression in wheat. This study addresses important aspects for the development of fungal‐derived resistance through the expression of silencing constructs in host plants as a powerful strategy to control cereal rust diseases.

## Introduction

Wheat stripe rust, caused by *Puccinia striiformis* f. sp. *tritici* (*Pst*), is one of the most serious diseases of wheat (*Triticum aestivum*) worldwide (Chen *et al*., 2013). This disease can result in more than 90% yield losses in a field (http://striperust.wsu.edu). The most economical, effective and environmentally friendly way of controlling this disease is to breed and use wheat varieties. However, most race‐specific host resistance genes have transient protection, probably due to the rapid evolution of new virulent rust fungal isolates (Fisher *et al*., 2012). Therefore, new feasible methods must be uncovered to protect wheat crops from rust fungi.

RNA interference (RNAi) is firstly discovered in *Caenorhabditis elegans* (Fire *et al*., 1998), and it can trigger potent and specific interference of pathogenic processes through exogenous double‐stranded RNA (dsRNA). For several insects and nematodes, silencing has been confirmed by merely feeding the dsRNA of the target genes (Hannon, 2002; Huvenne and Smagghe, 2010). With the identification of small RNAs (siRNAs), the mechanism of gene silencing of essential cellular functions was established (Castel and Martienssen, 2013; Vaucheret and Fagard, 2001). RNAi has been proved to be a powerful tool to reveal functions of target genes in organisms (Hellens *et al*., 2005) and offers an effective strategy to enhance resistance in crop plants. The accumulation of RNAi molecules in barley targeting fugal transcripts affects the development of *Blumeria graminis* f. sp. *hordei* in barley (Nowara *et al*., 2010). Transient silencing genes encoding MAP kinases, calcineurin B (PtCNB) and cyclophilin (PtCYC1), lead to increased resistance in wheat against rust diseases (Panwar *et al*., 2013a). Expressing inverted repeat fragments of cellulose synthase (CES1) genes of *Bremia lactucae* in transgenic plants resulted in attenuated pathogenicity and growth of *B. lactucae* (Govindarajulu *et al*., 2015). Three RNAi constructs derived from *Chs3b* were identified as the most effective RNAi constructs for enhancing resistance to *Fusarium* pathogens *in planta* (Cheng *et al*., 2015).

As a biotrophic parasite, *Pst* infects the host mainly from urediospores, which germinate within 3 h at a low temperature after deposition on the leaf surface (Hassebrauk and Schroeder, 1964). Germ tubes grow perpendicular to the long axis of epidermal cells of the leaf until they encounter a stoma. At 6–8 h postinoculation (h p.i.), an appressorium forms above the stoma and subsequently a substomatal vesicle forms within the stomatal cavity. At 12–18 h p.i., the primary infection hypha and haustorial mother cells emerge. Haustorial mother cells (HMCs), which have a thick, multilayered wall, invaginate the host cell plasma membrane with a slender neck to form the haustorium (Kang *et al*., 2003). Haustoria withdraw nutrients from host cells through the extrahaustorial matrix (Voegele and Mendgen, 2003). The primary infection hyphae branch and produce a number of HMCs and haustoria from 24 to 144 h p.i., developing into the fungal mycelium within the host tissue. From 6 to 8 days after infection, symptoms of chlorosis will be observed, whereas sporulation commences after approximately 12–14 days under favourable conditions.

The cyclic adenosine monophosphate protein kinase A (cAMP‐PKA) signalling pathway is well conserved across eukaryotes and has been proved to participate in virulence, morphogenesis and development in diverse fungi (Bahn and Sundstrom, 2001; D'Souza and Heitman, 2001; Fuller and Rhodes, 2012). In *Saccharomyces cerevisiae*, cAMP‐dependent protein kinase has a vital role in controlling proliferation, stress resistance, metabolism and the availability of nutrients (Thevelein and De Winde, 1999; Toda *et al*., 1987). The catalytic subunits of PKA including *Tpk1‐3* have different functions. *Tpk2* has a unique role in the activation of pseudohyphal growth. In contrast, *Tpk1* and *Tpk3* repress filamentation (Pan and Heitman, 1999; Robertson and Fink, 1998). Further studies revealed that *Tpk2* has a negative role in regulating genes for iron uptake, whereas it has a positive role in regulating genes for water homeostasis and trehalose degradation (D'Souza *et al*., 2001). In *Magnaporthe oryzae*,* CPKA* was shown to inhibit appressorium formation and the responsiveness of germinating conidia to exogenous cAMP (Mitchell and Dean, 1995). The maintenance of pathogenicity in *cpkA* mutants on wounded plants implies an additional role of *CPKA* that may be essential for appressorial penetration (Xu *et al*., 1997). In *Fusarium graminearum*,* CPK1* is responsible for hyphal growth, differentiation and pathogenesis (Hu *et al*., 2014). In *Ustilago maydis*, two genes *adr1* and *uka1* are found to encode catalytic subunits of PKA. The *adr1* is the major PKA catalytic subunit gene and required for pathogenicity, whereas *uka1* has almost no influence on pathogenicity (Dürrenberger *et al*., 1998). The availability of rust fungus genomic resources (Duplessis *et al*., 2011; Xu *et al*., 2011; Zheng *et al*., 2013) accelerates the prediction of a great number of genes and the research on functional genomics of rust fungi. However, due to the lack of functional genomics tools for rust fungi, less is known about biological functions of these genes.

In this study, we characterized a gene encoding the catalytic subunit of PKA, designated *PsCPK1* in *Pst*. The results showed that knockdown of *PsCPK1* leads to decreased virulence of *Pst*. The hairpin silencing constructs of *PsCPK1* expressed in wheat plants are sufficient to suppress disease development of *Pst*, indicating durable resistance at the genetic level against *Pst* infection.

## Results

### *Pst* contains two PKA catalytic subunit genes

**Figure 1:**
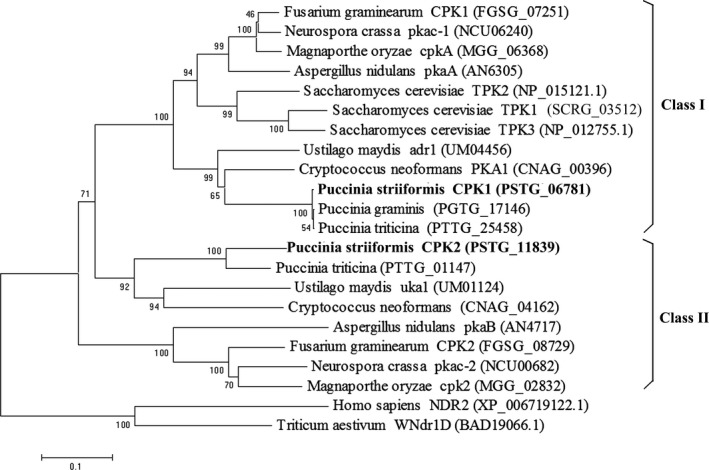
Phylogenetic analysis of the catalytic subunits of fungal cAMP‐dependent protein kinases. The amino acid sequences encoded by the catalytic subunits of the cAMP‐dependent protein kinases from *Puccinia striiformis* f. sp. *tritici* (PSTG), *P. triticina* (PTTG), *P. graminis* f. sp. *tritici* (PGTG), *Ustilago maydis* (UM), *Cryptococcus neoformans* (CNAG), *Fusarium graminearum* (FGSG), *Magnaporthe oryzae* (MGG), *Saccharomyces cerevisiae* (SCRG), *Neurospora crassa* (NCU) and *Aspergillus nidulans* (AN) were retrieved from the NCBI. Phylogenetic analysis was carried out with the MEGA6 software by the neighbour‐joining method.

A BLAST search using adr1 and uka1 of *U. maydis* (Dürrenberger *et al*., 1998) as queries revealed that the genome of *Pst* contains two PKA catalytic subunit genes, *PSTG06781* and *PSTG11839*, that were named *PsCPK1* and *PsCPK2*, respectively (Zheng *et al*., 2013). In this study, sequence analysis indicated that *PsCPK1* has an open reading frame (ORF) of 1443 bp, encoding a putative protein composed of 480 amino acids with a molecular weight of 55.69 kD and an isoelectric point (pI) of 6.72. A multisequence alignment with seven CPK proteins of different organisms in NCBI database revealed that *PsCPK1* is 80% and 57.3% identical to *CPK1* from *Puccinia graminis* f. sp. *tritici* (*Pgt*) and *Puccinia triticina* (*Pt*), respectively, and contain almost all the conserved domains of the PKA catalytic subunit. Compared to the PsCPK1 protein, PsCPK2 shares 30.57% similarity and 40.54% identity at the nucleotide sequence level. Phylogenetic analysis indicated that the homologs of *CPK* in *Pst* separate into two distinct groups, class I and class II (Figure 1). *PsCPK1* in class I is orthologous to *adr1* of *U. maydis*,* cpkA* of *M. oryzae* and yeast *TPK2*, whereas *PsCPK2* in class II is orthologous to *cpk2* of *M. oryzae* and *uka1* of *U. maydis*. These results indicate that *CPK* is highly conserved in other filamentous fungi.

### *PsCPK1* is highly expressed at the early infection stage of *Pst*

**Figure 2:**
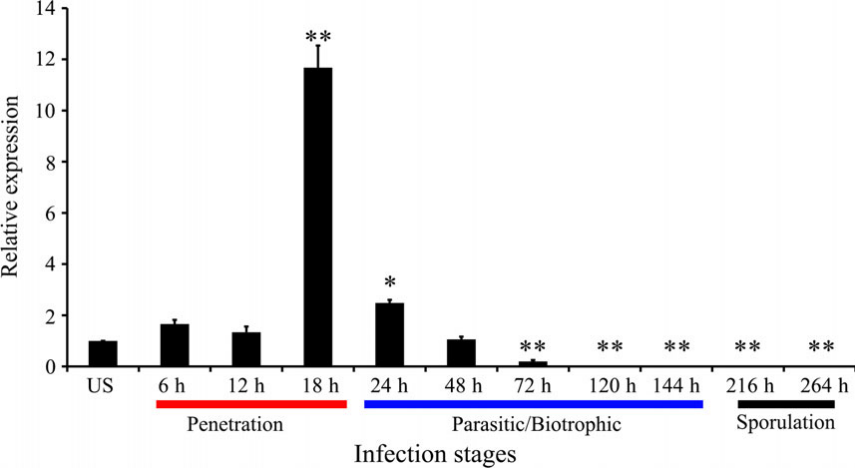
Transcript profiles of *PsCPK1* at different *Pst* infection stages. Wheat leaves inoculated with virulent *Pst* isolate CYR32 sampled at 0, 6, 12, 18, 24, 36, 48, 72, 120, 144, 216 and 264 h postinoculation (h p.i.). Relative expression of *PsCPK1* was calculated by the comparative threshold (2^−ΔΔ^ ^CT^) method. Mean and standard deviation were calculated with data from three independent biological replicates. Differences were assessed using Student's *t*‐tests. Asterisks indicate *P* < 0.05, and double asterisks indicate *P* < 0.01.

To investigate whether *PsCPK1* is involved in *Pst* infection, quantitative RT‐PCR (qRT‐PCR) was used to test *PsCPK1* transcript levels in different *Pst* infection stages. The transcript level of *PsCPK1* was gradually induced as early as 6 h p.i. and at 18 h p.i. attained the maximum level of 11.7‐fold compared with that in the control. Then, the transcript level returned to the original level at 24–48 h p.i. (Figure 2). Its expression down‐regulated between 72 and 264 h p.i. and was barely detected at the sporulation stage (216–264 h p.i.). Our results indicate that the transcription of *PsCPK1* is induced during the infection stage.

### *PsCPK1* partially complements the *M. oryzae cpkA* mutant

**Figure 3:**
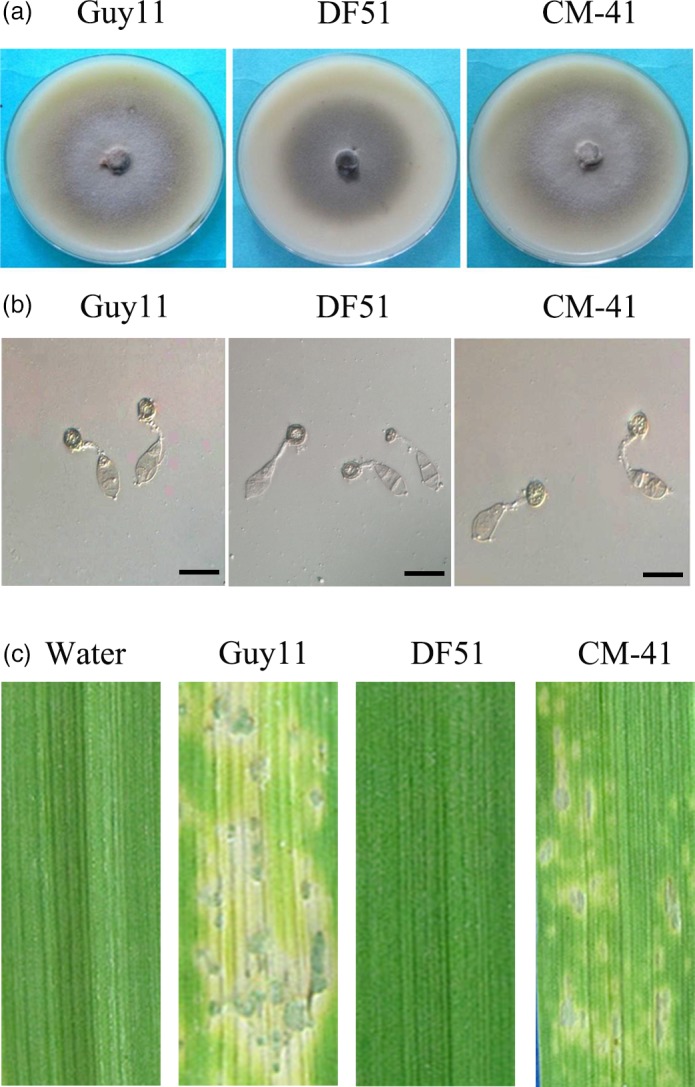
Complementation of the *cpkA* mutant with the *PsCPK1* fusion construct. (a) Colony morphology of *Magnaporthe oryzae* strains. Colonies of the wild‐type (Guy11), *cpkA* deletion mutant (DF51) and complemented strain (CM‐41) grown on PDA plates for 5 dpi. (b) Appressorium formation assay. Germ tubes from the wild‐type strain (Guy11) developed appressoria by 24 h p.i., but no appressorium formation was observed in the *cpkA* mutant DF51. Under the same conditions, a transformant of expressing the *PsCPK1* fusion construct (CM‐41) formed appressoria. Bar, 25 mm. (c) Barley infection assay. Left to right, barley leaves were sprayed with sterile water and conidia of Guy11, DF51 or CM‐41. Typical leaves were photographed at 6 dpi postinoculation.

The PsCPK1 protein shares 80% similarity and 66% identity with CPK1 of *M. oryzae*. To perform complementation analysis, we transformed the *PsCPK1* gene into the *cpkA* mutant DF51. The resultant transformants showed identical phenotypes and only transformant CM‐41 is used for subsequent analysis. On oatmeal agar medium plates, aerial mycelium of transformant CM‐41 was similar to that of Guy11 (the wild‐type strain), and almost no aerial mycelium was observed in the *cpkA* mutant DF51 (Figure 3a). These results indicate that the *PsCPK1* gene partially complements the defects of the *M. oryzae cpkA* mutant (DF51) in vegetative growth.

Further assays on appressorium formation and plant infection with the transformant CM‐41 were performed. The results showed that over 90% of the germ tubes formed appressoria by 24 h in Guy11. However, under the same conditions, approximately 50% of the germ tubes formed appressoria in CM‐41 and no appressoria was observed for the *cpkA* mutant DF51 (Figure 3b). To test the pathogenicity of the transformant CM‐41, eight‐day‐old barley seedlings of cultivar NB6 were sprayed with conidia of CM‐41. At 6 dpi, leaves inoculated with CM‐41 or Guy11 developed typical blast lesions, whereas fewer and smaller lesions were observed on leaves inoculated with CM‐41 (Figure 3c). No lesions were found on leaves sprayed with water or conidia of DF51 (Figure 3c). Our data show that *PsCPK1* can partially complement the DF51 in appressorium formation and plant infection.

### Transient silencing of *PsCPK1* significantly reduces pathogenicity of *Pst*

**Figure 4:**
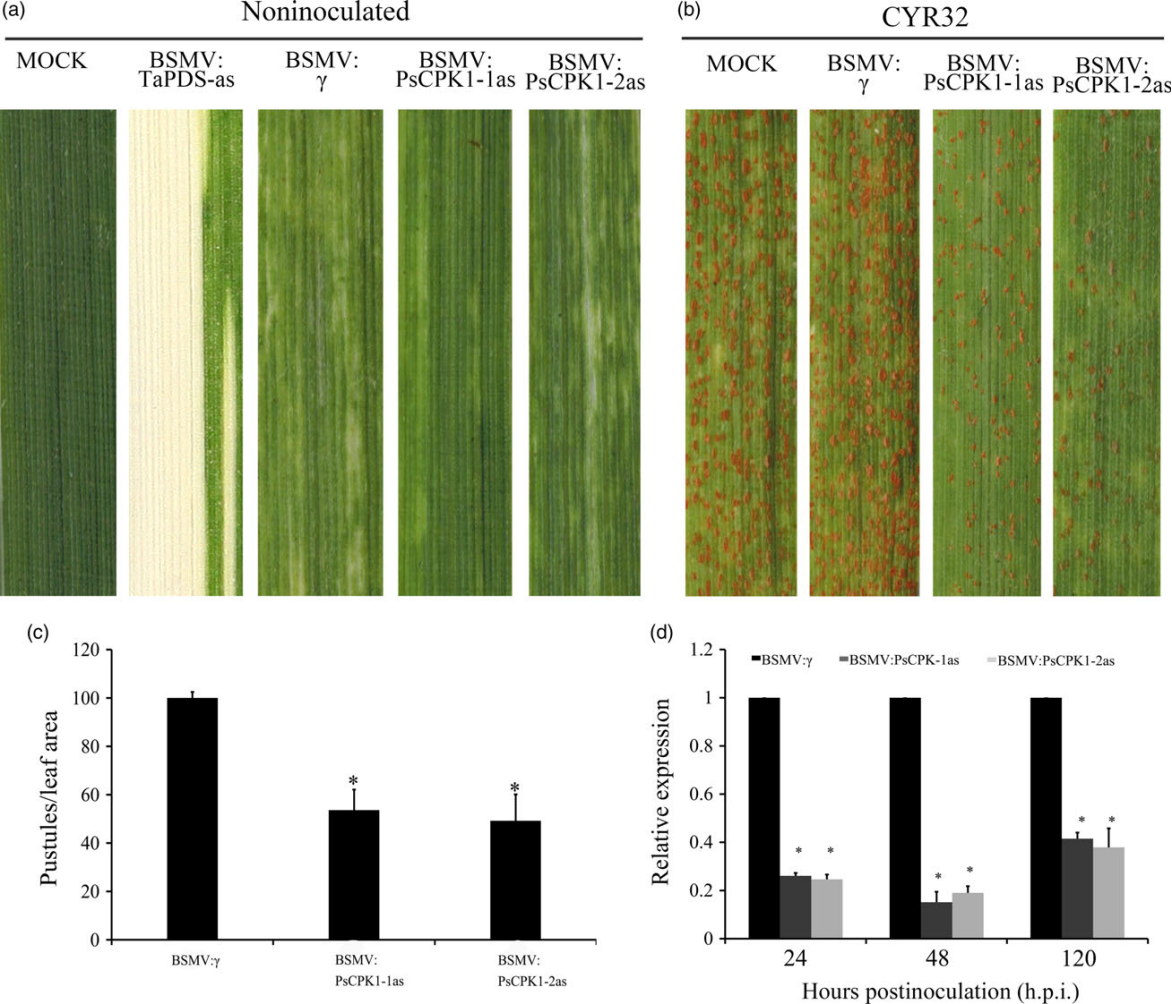
Functional assessment of *PsCPK1* in *Pst* pathogenicity determined by BSMV‐mediated HIGS. (a) Mild chlorotic mosaic symptoms were observed on the fourth leaves of wheat seedlings inoculated with BSMV: γ (control), BSMV:PsCPK1‐1as and BSMV:PsCPK1‐2as. No change in phenotype was observed in wheat leaves mock‐inoculated with FES buffer (MOCK). Photobleaching was evident on wheat leaves infected with BSMV:TaPDS‐as. (b) Phenotypes of the fourth leaves of BSMV:γ (control)‐, BSMV:PsCPK1‐1as‐ and BSMV:PsCPK1‐2as‐inoculated wheat plants 14 dpi with *Pst* isolate CYR32. (c) Quantification of the uredial density in the BSMV:γ‐, BSMV:PsCPK1‐1as‐ and BSMV:PsCPK1‐2as‐inoculated wheat plants 14 dpi with *Pst* isolate CYR32. (d) Relative transcript levels of *PsCPK1* in the BSMV:γ‐, BSMV:PsCPK1‐1as‐ and BSMV:PsCPK1‐2as‐inoculated wheat plants 24, 48 and 120 h p.i. with *Pst* isolate CYR32. Values are expressed relative to the endogenous *Pst* reference gene *EF1*, with the empty vector (BSMV:γ) set at 1. Values represent the means ± standard error of three independent assays. Differences were assessed using Student's *t*‐tests, and asterisks indicate *P* < 0.05.

BSMV‐mediated HIGS was adopted to silence *PsCPK1* in *Pst*. Two specific fragments of *PsCPK1* were designed for silencing this gene (Figure S1). Ten days after infection with BSMV, wheat seedlings inoculated with sterile FES buffer developed normal leaves (Figure 4a). Under the same conditions, photobleaching was observed in BSMV:TaPDS‐as infected plants (Figure 4a), indicating that *TaPDS* was successfully and specifically silenced. All wheat seedlings inoculated with BSMV:γ (control), BSMV:PsCPK1‐1as and BSMV:PsCPK1‐2as showed mild chlorotic mosaic symptoms on the third leaf (Figure 4a), suggesting that the BSMV‐HIGS system functioned well. In comparison with the control plants, wheat leaves infected with BSMV:PsCPK1‐1as and BSMV:PsCPK1‐2as displayed a susceptible phenotype with similar viral infection symptoms (Figure 4b). However, the number of uredia in BSMV:PsCPK1‐1as‐ and BSMV:PsCPK1‐2as‐infected leaves was significantly lower than that in the control (Figure 4b). Compared with control plants, the ratio of pustules on leaves infected with BSMV:PsCPK1‐1as and BSMV:PsCPK1‐2as was reduced by 49% and 54%, respectively (Figure 4c). To test silencing efficiency of BSMV‐HIGS, qRT‐PCR was used to assay the relative transcript level of *PsCPK1* at 24, 48 and 120 h p.i. with the virulent CYR32 isolate. Compared with control plants, the transcript level in the BSMV:PsCPK1‐1as‐infected leaves was reduced by 74%, 85% and 58%, respectively (Figure 4d). Similarly, in BSMV:PsCPK1‐2as‐infected leaves, the transcript level of *PsCPK1* was reduced by 75%, 81% and 62%, respectively (Figure 4d).

The detailed histological changes in HIGS‐silenced plants inoculated with *Pst* CYR32 were microscopically examined (Figure S2a–f). In BSMV:PsCPK1‐1as‐ and BSMV:PsCPK1‐2as‐inoculated leaves, the hyphal lengths exhibited significant reduction relative to that in the control plants at 48 and 120 h p.i. (Figure S2g). The infection areas in the silenced plants also decreased at 120 h p.i. (Figure S2h). However, the numbers of haustorial mother cells showed no significant difference at 48 h p.i. (Figure S2i).

### Molecular analysis of transgenic wheat plants

**Figure 5:**
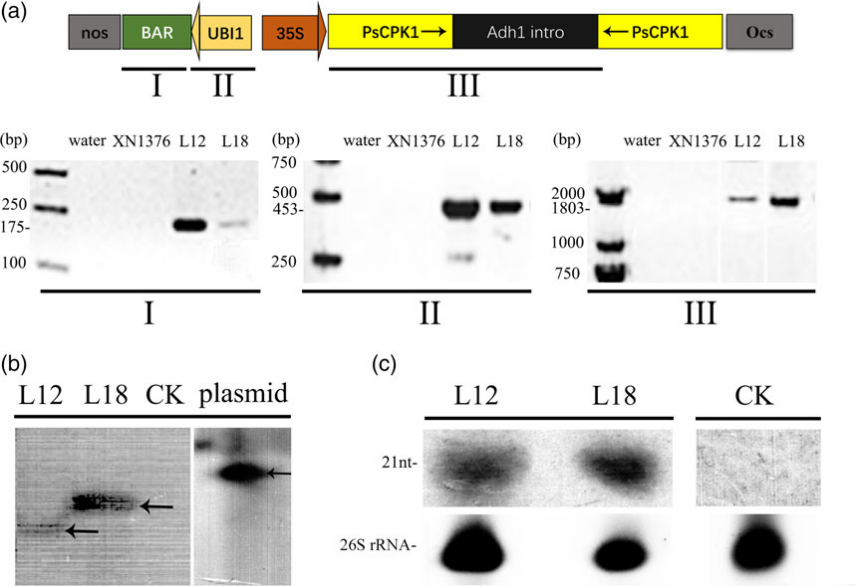
Molecular analysis of the *Pst*‐resistant transgenic wheat plants. (a) Diagram showing the RNAi cassette in the construct pMCG161‐RNAi for wheat transformation. T_4_ plants were analysed by genomic PCR for the presence of the selectable marker gene bar (I), *UBI1* promoter (II) and fragment (*PsCPK1‐intro*) of the RNAi cassette (III). The III image was compiled from different pictures. (b) Southern blotting analysis of the transgenic plants. Genomic DNA isolated from control plants (CK), T_4_ transgenic plants (L12 and L18) and plasmid pMCG161‐PsCPK1‐RNAi. DNAs were digested with *Bam* HI and *Xho*I and hybridized to the hairpin fragment probe. The image was compiled from the same picture which we removed the lanes indicating other T_4_ transgenic lines. (c) The expression of the small RNA in T_4_ generation lines was analysed by RNA gel blotting. 26S rRNA, loading/blotting control. RNA blots were hybridized with the corresponding target gene‐specific probes. siRNA levels in plants silenced using the pMCG161‐PsCPK1‐RNAi DNA vector expressing target fungal fragments with a mixture of sense plus antisense forms (L12 and L18). No signal was detected in the transgenic lines carrying empty vector (CK). Total RNA was extracted from three different plants. Lower panels, ethidium bromide‐stained rRNA as gel loading controls. The arrowhead indicates an oligonucleotide marker of 21 nucleotides.

To efficiently generate specific siRNAs directed against the *PsCPK1* gene in transgenic wheat plants, the coding region of *PsCPK1* (1443 bp) was cloned into the plasmid pMCG161 and the cloned fragments as inverted repeats under control of the maize ubiquitin promoter (*Ubi1*) to generate the dsRNA (Figure 5a). The resultant construct was then bombarded into wheat cultivar (cv.) Xinong1376 (XN1376). In the glasshouse experiments, ten transgenic lines (L1, L2, L5, L9, L10, L11, L12, L14, L17 and L18) (Table S1) were tested and the disease severity scale was evaluated by the standard described in Table S2 (Line and Qayoum, 1992). Of these ten lines, the transgenic L12 and L18 lines were selected for further study (Figure S3). Integration of the transgene in the two lines L12 and L18 carrying the resultant constructs was confirmed by genomic PCR with primers Bar‐F/R, UBI1‐F/R and TG‐CPK1‐F/R (Table S3; Figure 5a). To further verify that these two lines were independently derived and transgenic, Southern blotting was performed with genomic DNA from the two lines digested with *Bam*HI and *Xho*I. The results showed that each line contained one copy and exhibited a different banding pattern (Figure 5b). To confirm whether HIGS efficiently works to protect transgenic wheat plants from *Pst* infection, northern blotting was performed to detect *PsCPK1* dsRNA expression in L12 and L18. The results show that L12 and L18 produce the small interfering RNAs (siRNAs, ~21 nt) (Figure 5c; Figure S4).

### Expression of *PsCPK1* small RNA in wheat confers durable resistance to *Pst*

**Figure 6:**
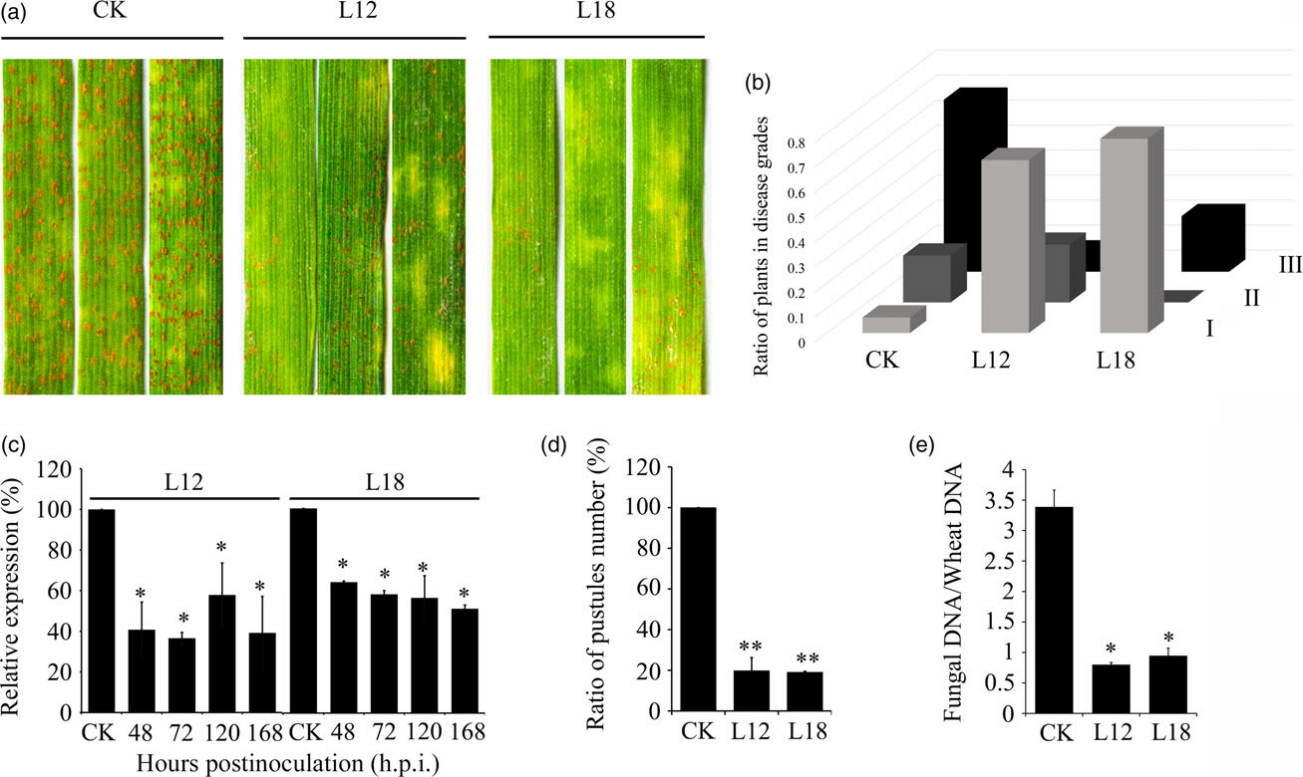
Expression of *PsCPK1* small RNA in wheat confers durable resistance to *Pst*. (a) Phenotypes of the second leaves of the third‐ and the fourth‐generation wheat plants at 14 dpi with *Pst* isolate CYR32. (b) The ratio of plants in disease grades. Phenotypes were scored to indicate the frequency of symptoms, as follows: I: 1–3; II: 4–6; III: 7–9. (c) Relative transcript levels of *PsCPK1* in the second leaves of the fourth‐generation wheat plants at 48, 72, 120 and 168 h p.i. with *Pst* isolate CYR32. Values are expressed relative to the endogenous *Pst* reference gene *PsEF1*, with the empty vector (CK) set at 1. Values represent the means ± standard error of three independent samples. (d) Quantification of the uredial density in the CK‐, L12‐ and L18‐inoculated wheat plants at 16 dpi. (e) Q‐PCR measurement of fungal biomass. Ratio of fungal to wheat nuclear genomes using fungal *PsEF1* and wheat *TaEF1* genes, respectively, in plants treated with variants targeting fungal genes compared with controls. Genomic DNA extracted from the second leaf from three different plants at 7 dpi. Values represent the means ± standard error of three independent samples. Differences were assessed using Student's *t*‐tests. Asterisks indicate *P* < 0.05, and double asterisks indicate *P* < 0.01.

*Pst* resistance was evaluated in the glasshouse. Average reduction in disease severity in L12 and L18 was 59% and 46%, respectively (Figure S3). Phenotypically, the second leaves of 14‐day‐old L12 and L18 plants were challenged with the virulent CYR32 isolate (Figure 6a). All of the infected leaves could be separated into three classes (Table S2), and the ratio of diseased plants in the 1–3 grade was 69% and 77% in L12 and L18 (Figure 6b). To determine the transcript levels of *PsCPK1* at different infection stages of *Pst* on XN1376, we performed qRT‐PCR assay (Figure S5). According to the results, we isolated total RNA from silenced leaves after 48, 72, 120 and 168 h p.i. with *Pst* urediospores to test silencing efficiency of *PsCPK1*. Relative expression analysis of these samples compared with transgenic controls revealed specific reductions in transcript levels of the *CPK1* gene in *Pst*. The transcript levels of the *PsCPK1* showed an approximate 42% to 64% down‐regulation in L12 and 36% to 50% in L18 compared with controls (Figure 6c), suggesting that *PsCPK1* suppression had an approximate relevance to increased resistance against *Pst* in L12 and L18. Additionally, a series of experiments provided evidence of the effectiveness and stability of the transgenes. To determine the ratio of sporulating to nonsporulating uredia, a quantitative assay was made within a defined surface area of the leaves at 16 days after *Pst* infection, which is consistent with the rust disease phenotype. The ratio of sporulating to nonsporulating uredia was significantly reduced by about 80% in transgenic lines. Fungal biomass in the infected leaves was also measured. Total genomic DNA was isolated from wheat leaves infected with *Pst*, and the relative levels of *PsEF1* and *TaEF1* were quantified by Q‐PCR (Figure S6). Compared with controls, fungal biomass was significantly reduced by about 76% in L12 and 72% in L18 at 7 dpi, respectively (Figure 6e). Together, these results indicate that in transgenic wheat plants *PsCPK1* expression is efficiently down‐regulated and *Pst* growth is impaired and delayed.

### Histological and molecular changes in *Pst* growth in transgenic plants

**Figure 7:**
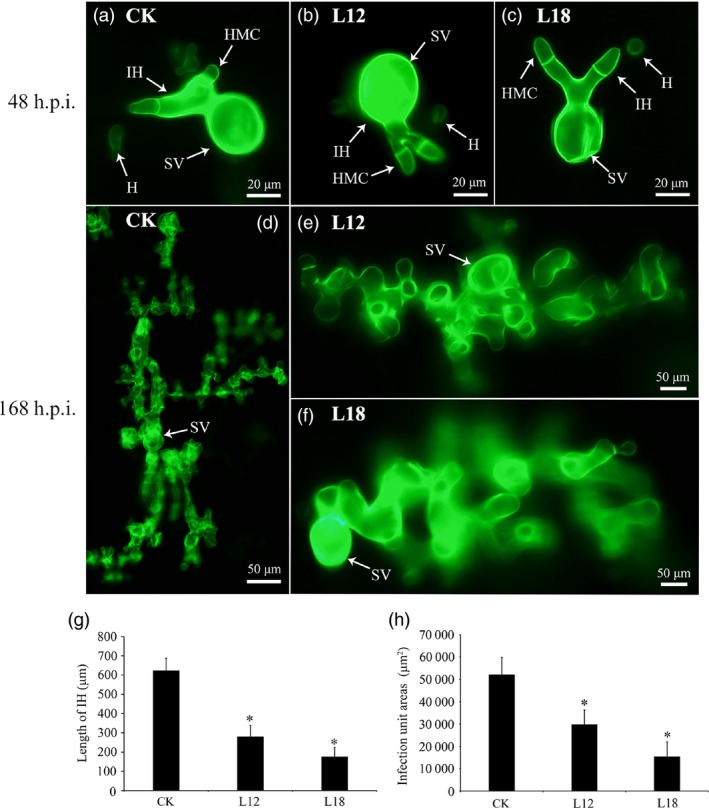
Histological changes in *Pst* growth in transgenic plants. (a–f) A microscopic examination revealed no obvious differences in the number of hyphal branches between the control plants (CK) and the transgenic plants (L12 and L18) at 48 h p.i. (a–c) and 168 h p.i. (d–f), respectively. (g) The hyphal lengths in the transgene lines (L12 and L18) were shorter than those observed in the control plants (CK) at 168 h p.i. (h) The colony sizes in the two transgenic plants (L12 and L18) were reduced compared with the sizes observed in the control plants (CK) at 168 h p.i. Differences were assessed using Student's *t*‐tests, and asterisks indicate *P* < 0.05.

**Figure 8:**
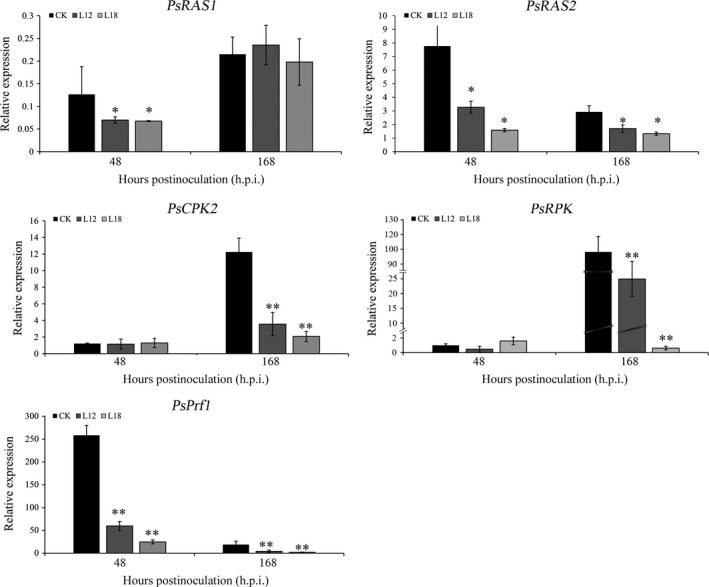
Transcript levels of some selected genes involved in cAMP‐PKA pathway of *Pst* after *Pst* infection. *PsRAS1*, GTPase Ras1; *PsRAS2*, GTPase Ras2; *PsCPK2*, catalytic subunits 2 of PKA;* PsRPK*, regulatory subunit of PKA;* PsPrf1*, HMG box transcription factor. Values represent the means ± standard error of three independent assays. Differences were assessed using Student's *t*‐tests, and asterisks indicate *P* < 0.05, and double asterisks indicate *P* < 0.01.

To further test the possible function of the *PsCPK1* in *Pst*–wheat interaction, we made microscopic assessment of *Pst* development in foliar tissue. The infection sites were chosen randomly from the second infected leaves of L12 and L18 plants and transgenic control plants at 48 and 120 h p.i. and analysed by fluorescence microscopy. At 48 h p.i., fungal penetration and expansion abilities were not different, and the mycelial morphology appeared normal compared with that in the transgenic control lines (Figure 7a–c). Mycelial morphology appeared normal (Figure 7d–f), whereas at 168 h p.i. both the length of infection hyphae (IH) (Figure 7g) and area of infection unit were significantly reduced (Figure 7h). There were no significant differences in the number of haustorial mother cells at 48 and 168 h p.i. (data not shown). To further understand the relationship between transgenic wheat and controls, we assayed the transcript profiles of a few selected genes after infection with *Pst*. The transcript levels of *PsRAS2,* upstream of the cAMP‐PKA pathway in *Pst*, were down‐regulated in transgenic lines compared with the control (Figure 8), but *PsRAS1* was only down‐regulated at 48 h p.i. We also subsequently monitored the relative expression of several other subunit genes of the PKA complex (*PsCPK2* and *PsRPK*) in transgenic plants (Figure 8). Interestingly, both were down‐regulated at 168 h p.i., but showed no obvious changes at 48 h p.i. (Figure 8). *PsPrf1,* a transcription factor functioning after phosphorylation by PsCPK1*,* decreased significantly in the L12 and L18 lines at 48 and 168 h p.i. Therefore, our data indicate that *PsCPK1* most likely plays an important role in the virulence of *Pst* by participating in fungal development and growth, and silencing of the *PsCPK1* results in virulence penalty of *Pst* in transgenic plants.

## Discussion

To compensate for the limited amount of germplasm resources and rapid loss of resistance to fungal diseases, several new approaches have emerged in recent years. Many previous studies have experimentally validated that RNAi is a promising approach for durable control of pathogenic fungi (Chen *et al*., 2016; Cheng *et al*., 2015; Ghag *et al*., 2014; Nowara *et al*., 2010). In the rust fungi, the RNAi approach has been developed through BSMV (Yin *et al*., 2011). The *Agrobacterium tumefaciens*‐mediated transient RNAi assay has been developed in wheat to target *P. triticina* pathogenicity genes (Panwar *et al*., 2013a). But, complete resistance has rarely been achieved in wheat, and it is therefore significant that transgenic resistance to *Pst* was identified to the fourth generation in our study. To our knowledge, this is the first reported transgenic wheat with durable disease resistance against *Pst*.

A few studies have proved that expression of dsRNA targeted at fungal genes in wheat leads to increased resistance against phytopathogenic fungi (Chen *et al*., 2016; Cheng *et al*., 2015). Usually, true transgenic lines in hexaploid wheat are available until the T_3_ generation (Lee *et al*., 2012), and then, we generated T_4_ generation for further investigation. By analysing the T_4_ generation, we confirmed by northern blot analysis that transgene‐derived siRNAs accumulate in the resistant transgenic plants. Moreover, those plants had the strongest reduction in mRNA and corresponding decrease in susceptibility. Southern blot analysis indicated that each resistant transgenic line contains a single copy derived from independent transformations. Unlike the resistance of germplasm resources, the reduction in the disease phenotype does not depend on race‐specific resistance (Johnson, 1988) largely because of the high conservation of *PsCPK1* in most isolates (data not shown), including the popular isolates CYR32 and V26 (Huang *et al*., 2014; Ren *et al*., 2015; Yin *et al*., 2009). Moreover, we found that transient silencing by BSMV‐HIGS is maintained for only 2 weeks, as indicated by Yin *et al*. (2011) and Miller *et al*. (2012), whereas the transgenic plants show a slow but continuous weakening of *Pst* silencing. Our results suggest that transgenic wheat has the potential to reduce the severity of stripe rust of wheat.

To further understand the mechanism after silencing the *PsCPK1*, we investigated the transcript levels of some important genes in the cAMP‐PKA pathway and the other subunit genes of the PKA complex. Ras proteins function at upstream of mitogen‐activated protein kinase (MAPK) or cAMP‐PKA pathway (Bluhm *et al*., 2007; Park *et al*., 2006). In *Pst*,* PsRas2* is required for *Pst* pathogenicity, but not for *PsRas1* (Cheng *et al*., 2016). *PsRas2* and *PsRas1* were significantly reduced after *PsCPK1* silencing, perhaps through the feedback loop of the cAMP (Figure S7). The genes *PsCPK2* and *PsRPK*, encoding another catalytic subunit and the regulatory subunit of the PKA complex, were down‐regulated at 168 h p.i. Interaction of the catalytic subunits of PKA with the regulatory subunit of PKA was activated by cAMP. Silencing of *PsCPK1* may destroy the structure of the PKA complex. As *Prf1* is required for cell fusion and filamentous growth (Hartmann *et al*., 1999) and the activity of Prf1 is controlled by PKA phosphorylation (Zarnack *et al*., 2008), a domino effect may appear in the transgenic plants after silencing of the *PsCPK1*. Together with these results, *PsCPK1* plays important roles in the cAMP‐PKA pathway and silencing the *PsCPK1* will disrupt the chain of cAMP‐PKA pathway after infection by *Pst* (Figure S7).

In the process of the application of RNAi methods in organisms, the off‐target effects maybe occur when the siRNA is partially complementary to one or more cellular mRNAs except the target (Jackson *et al*., 2003; de Souza, 2014). Despite the high conservation of *CPK1* in different fungi, the *PsCPK1* is sequence specific among its homologues in pathogenic fungi at the nucleotide level. Additionally, the homologous *PsCPK1* gene in wheat shares up to 30% identity at the nucleotide sequence level within the coding regions. The possibility of off‐target effects was analysed by Si‐Fi software and no effective hits were found (Table S4). These off‐target effects can show no obvious difference in the growth of wheat (Figure S8). Furthermore, the off‐target effect could be more pronounced in rust fungi, which might have resulted in the disease suppression.

HIGS has been proved to be a novel tool to reveal gene function in obligate biotrophic fungus (Panwar *et al*., 2013a; Yin *et al*., 2011) and offers the potential of disease control (Nowara *et al*., 2010). In this study, wheat plants expressing the RNAi constructs showed strong and genetically stable resistance to *Pst* in the fourth generation. Our results indicated that *PsCPK1* is an excellent target to generate durable genetic resistance against *Pst* and provides a potential reservoir of novel resistance resources of wheat against rust fungi. But the application of this material in agricultural production obviously requires more field trials. With the rapid development of various sequencing technologies, quantitative proteomics and RNA‐seq analysis may be used in this material to understand the mechanism of the cAMP‐mediated signal transduction pathway. The improved understanding will guide the development of genetic breeding, which could contribute to environmentally sustainable agriculture.

## Experimental procedures

### Biological materials, growth condition, fungal inoculation

The wheat cv. Suwon11 (Su11) and the *Pst* virulent isolate CYR32 were used in this study (Guo *et al*., 2011). *Pst* isolate CYR32 was maintained and propagated on susceptible wheat cv. Mingxian169. The germplasm used for gene transformation was XN1376, a high‐yielding and early‐maturing winter wheat variety. Plant cultivation and fungal inoculation were carried out following the procedures and conditions previously described (Kang *et al*., 2002).

### Plasmid construction and plant transformation

To generate pMCG161‐CPK1, the PCR was performed to obtain the product with the primers PsCPK1‐F and PsCPK1‐R (Table S3). The amplified fragment was subsequently cloned into the *Asc*I and *Avr*II and *Asi*SI and *Spe*I sites of plasmid pMCG161. Then, the CPK1+‐intro‐CPK1‐gene from T‐simple (TaKaRa, Tokyo, Japan) was cut with *Asc*I and *Avr*II and ligated into the pMCG161 with *Asc*I and *Avr*II.

### Sequence alignments and polymorphism analysis

One cDNA clone encoding *PsCPK1* (GenBank accession no. KY346510)was designed with special primer CPK1‐F/R (Table S3) according to the sequence from the cDNA library during wheat–*Pst* interaction (Ma *et al*., 2009). *PsCPK1* sequence was firstly analysed using BLAST search and ORF Finder, and then, the conserved domain of PsCPK1 was detected with InterProScan and ScanProsite (Guo *et al*., 2013). Multiple sequence alignment was implemented with DNAMAN6.0 (Lynnon BioSoft, Quebec, Canada) and CLUSTALX2.0 (Chenna *et al*., 2003). The phylogenetic tree was constructed with the Mega 6.0 software (Tamura *et al*., 2013).

### RNA extraction, cDNA synthesis and qRT‐PCR

To evaluate the transcript levels of *PsCPK1* in response to *Pst* infection, wheat leaves were sampled at 0, 6, 12, 18, 24, 36, 48, 72, 120, 144, 216 and 264 h p.i. according to previous microscopic observations of wheat–*Pst* interaction (Wang *et al*., 2007). To determine the efficiency of BSMV‐mediated HIGS, the fourth leaves of *PsCPK1*‐knockdown plants were sampled at 24, 48 and 120 h p.i. with *Pst*. Total RNA was isolated with the Trizol reagent (Invitrogen, Carlsbad, CA), and the first‐strand cDNA was synthesized for qRT‐PCR. qRT‐PCR was performed with a 7500 Real‐Time PCR System (Applied Biosystems, Foster City, CA), and PCR conditions were used as previously described (Liu *et al*., 2012). The *Pst* translation elongation factor 1 (*PsEF1*) gene was used as the internal reference for normalization (Guo *et al*., 2011). The transcript levels of *PsCPK1* and other genes in this study were assayed by the comparative 2^−ΔΔCT^ method (Livak and Schmittgen, 2001).

### Complementation of the *M. oryzae cpkA* mutant with *PsCPK1*

To perform complementation assays, the full length of the *PsCPK1* gene was obtained with primers CPK1‐CM‐F and CPK1‐CM‐R (Table S3). Then, the *PsCPK1* gene was cotransformed with the vector pFL2 into *S. cerevisiae* strain XK1‐25 to achieve plasmid pMoPsCPK1. The plasmid pMoPsCPK1 was transformed into protoplasts of the *M. oryzae cpkA* mutant DF51. To confirm the *PsCPK1* gene integrated into the *M. oryzae* genome, we isolated the resultant transformants and verified them by PCR with primers CPK1‐CM‐F and CPK1‐CM‐R. The assays for appressorium formation and plant infection were performed as previously described (Guo *et al*., 2011).

### BSMV‐mediated *PsCPK1* gene silencing

To further determine the role of *PsCPK1* during *Pst* infection, the *PsCPK1* gene was silenced with the BSMV‐HIGS system. To make sure the specificity for *PsCPK1* silencing, a 224‐bp fragment of *PsCPK1* in the 5′UTR named *PsCPK1*‐1as and a 296‐bp fragment of *PsCPK1* in the 3′UTR named *PsCPK1*‐2as were cloned with primers Higs‐CPK1‐1as‐F, Higs‐CPK1‐1as‐R, Higs‐CPK1‐2as‐F and Higs‐CPK1‐2as‐R (Table S3) and inserted into the virus plasmid. The BSMV RNAs were prepared *in vitro* from linearized plasmid γ‐TaPDSas, γ‐*PsCPK1*‐1as, γ‐*PsCPK1*‐2as, γ, α, β using the Message T7 *in vitro* transcription kit (Ambion, Austin, TX). The wheat leaves were used for inoculation with BSMV according to the procedures as previously described (Guo *et al*., 2013; Scofield *et al*., 2005). After inoculation with BSMV at the second leaf stage, wheat seedlings were maintained in a growth chamber at 23 ± 2 °C and examined for symptoms. In all experiments, the recombinant virus BSMV:TaPDSas was applied as a positive control. When the photobleaching phenotype was observed, the fourth leaves of *PsCPK1* silencing group were inoculated with urediospores of *Pst* isolate CYR32. The resistant or susceptible phenotypes were visible at 15 dpi.

### Histological observations of *Pst* growth and wheat response

The wheat leaves inoculated with BSMV at 24, 48 and 120 h p.i. and transgenic wheat leaves at 48 and 168 h p.i. were collected for histological observation as previously described (Guo *et al*., 2013). Stained leaf segments were fixed and cleared in ethanol/acetic acid (1:1 v/v). Autofluorescence of attacked mesophyll cells was observed as a necrotic death area with the Olympus BX‐51 microscope (Olympus, Tokyo, Japan). Infection sites and lengths of infection hyphae were measured under the blue light excitation. Fifty infection sites were examined on each randomly selected leaf segment per treatment for the measurement of fungal structures.

### Northern blotting analysis

Northern blotting was used to detect the accumulation of small RNAs as described previously (Zheng *et al*., 2007). The total RNA from 12‐day‐old seedlings was extracted using Trizol reagent (Invitrogen). The poly(ethylene glycol) enrichment method was to obtain small RNA (Zheng *et al*., 2007). The random priming method was used to label the probe for the detection of mRNA transcripts. The primers NB‐CPK1‐F and NB‐CPK1‐R used to obtain the probe are listed in Table S3.

### Southern blotting analysis

A CTAB‐based method was used to isolate total genomic DNA from 12‐day‐old seedlings (Hormaza, 2002). Aliquots (20 μg) of genomic DNA were digested overnight at 37 °C with *Bam*HI and *Xho*I, fractionated in 0.8% (w/v) agarose gel and blotted onto a nylon membrane (Biotrace, Gelman Sciences, Ann Arbor, MI) according to the protocols previously described (Sambrook and Russell, 1989). The 302‐bp cDNA fragment was obtained by RT‐PCR with primer SB‐CPK1‐F/R (Table S3) and then used as the probe. The membrane was hybridized with the probe labelled with [α‐^32^P] dCTP. Hybridization, posthybridization washes and signal detection were carried out as described for northern blotting (Sambrook and Russell, 1989), but the hybridization temperature was adjusted to 65 °C.

### Statistical analysis

Statistical testing was performed with the statistical software version package of IBM SPSS Statistics 21 (IBM SPSS Statistics, IBM Corporation, Armonk, NY). The data were tested by Student's *t*‐test (*P* < 0.05 or *P* < 0.01).

## Supporting information


**Figure S1** Amino acid sequence alignments of PsCPK1 with other fungal catalytic subunits of PKA.

**Figure S2** Histological observation of fungal growth in *PsCPK1*‐knockdown wheat plants after inoculation with *Pst* isolate CYR32.

**Figure S3** Bioassay of the transgenic wheat plants for *Pst* resistance.

**Figure S4** The expression of the small RNA in T_4_ generation lines was analysed by RNA gel blotting.

**Figure S5** Transcript profiles of *PsCPK1* in the leaves of wheat cultivar XN1376 infected by *Pst* isolate CYR32.

**Figure S6** Standard curves generated for the absolute quantification of *Pst* (A) and wheat (B).

**Figure S7** Schematic presentation of possible HIGS mechanisms involved in PKA pathway.

**Figure S8** No significant difference was observed for the growth between transgenic and control wheat lines.

**Table S1** Transformation pipeline and efficiency for producing transgenic lines used in this study.

**Table S2** Disease severity scale of wheat stripe rust.

**Table S3** Primers designed for *PsCPK1* research.

**Table S4** Prediction off‐target transcripts of *PsCPK1* gene.

## References

[pbi12829-bib-0001] Bahn, Y. and Sundstrom, P. (2001) *CAP1*, an adenylate cyclase‐associated protein gene, regulates bud‐hypha transitions, filamentous growth, and cyclic AMP levels and is required for virulence of *Candida albicans* . J. Bacteriol. 183, 3211–3223. 11325951 10.1128/JB.183.10.3211-3223.2001 PMC95223

[pbi12829-bib-0002] Bluhm, B.H. , Zhao, X. , Flaherty, J.E. , Xu, J. and Dunkle, L.D. (2007) RAS2 regulates growth and pathogenesis in *Fusarium graminearum* . Mol. Plant Microbe Interact. 20, 627–636. 17555271 10.1094/MPMI-20-6-0627

[pbi12829-bib-0003] Castel, S.E. and Martienssen, R.A. (2013) RNA interference in the nucleus: roles for small RNAs in transcription, epigenetics and beyond. Nat. Rev. Genet. 14, 100–112. 23329111 10.1038/nrg3355 PMC4205957

[pbi12829-bib-0004] Chen, W. , Wellings, C. , Chen, X. , Kang, Z. and Liu, T. (2013) Wheat stripe (yellow) rust caused by *Puccinia striiformis* f. sp. *tritici* . Mol. Plant Pathol. 15, 433–446. 10.1111/mpp.12116 PMC6638732 24373199

[pbi12829-bib-0005] Chen, W. , Kastner, C. , Nowara, D. , Oliveira‐Garcia, E. , Rutten, T. , Zhao, Y. , Deising, H.B. *et al* (2016) Host‐induced silencing of *Fusarium culmorum* genes protects wheat from infection. J. Exp. Bot. 67, 4979–4991. 27540093 10.1093/jxb/erw263 PMC5014151

[pbi12829-bib-0006] Cheng, W. , Song, X. , Li, H. , Cao, L. , Sun, K. , Qiu, X. , Xu, Y. *et al* (2015) Host‐induced gene silencing of an essential chitin synthase gene confers durable resistance to *Fusarium* head blight and seedling blight in wheat. Plant Biotechnol. J. 13, 1335–1345. 25735638 10.1111/pbi.12352

[pbi12829-bib-0007] Cheng, Y. , Wang, W. , Yao, J. , Huang, L. , Voegele, R.T. , Wang, X.J. and Kang, Z.S. (2016) Two distinct *Ras* genes from *Puccinia striiformis* exhibit differential roles in rust pathogenicity and cell death. Environ. Microbiol. 18, 3910–3922. 27206348 10.1111/1462-2920.13379

[pbi12829-bib-0008] Chenna, R. , Sugawara, H. , Koike, T. , Lopez, R. , Gibson, T.J. , Higgins, D.G. and Thompson, J.D. (2003) Multiple sequence alignment with the Clustal series of programs. Nucleic Acids Res. 31, 3497–3500. 12824352 10.1093/nar/gkg500 PMC168907

[pbi12829-bib-0009] D'Souza, C.A. and Heitman, J. (2001) Conserved cAMP signaling cascades regulate fungal development and virulence. FEMS Microbiol. Rev. 25, 349–364. 11348689 10.1111/j.1574-6976.2001.tb00582.x

[pbi12829-bib-0010] D'Souza, C.A. , Alspaugh, J.A. , Yue, C. , Harashima, T. , Cox, G.M. , Perfect, J.R. and Heitman, J. (2001) Cyclic AMP‐dependent protein kinase controls virulence of the fungal pathogen *Cryptococcus neoformans* . Mol. Cell. Biol. 21, 3179–3191. 11287622 10.1128/MCB.21.9.3179-3191.2001 PMC86952

[pbi12829-bib-0011] Duplessis, S. , Cuomo, C.A. , Lin, Y. , Aerts, A. , Tisserant, E. , Veneault‐Fourrey, C. , Joly, D.L. *et al* (2011) Obligate biotrophy features unraveled by the genomic analysis of rust fungi. Proc. Natl Acad. Sci. USA, 108, 9166–9171. 21536894 10.1073/pnas.1019315108 PMC3107277

[pbi12829-bib-0012] Dürrenberger, F. , Wong, K. and Kronstad, J.W. (1998) Identification of a cAMP‐dependent protein kinase catalytic subunit required for virulence and morphogenesis in *Ustilago maydis* . Proc. Natl Acad. Sci. USA, 95, 5684–5689. 9576944 10.1073/pnas.95.10.5684 PMC20439

[pbi12829-bib-0013] Fire, A. , Xu, S. , Montgomery, M.K. , Kostas, S.A. , Driver, S.E. and Mello, C.C. (1998) Potent and specific genetic interference by double‐stranded RNA in *Caenorhabditis elegans* . Nature, 391, 806–811. 9486653 10.1038/35888

[pbi12829-bib-0014] Fisher, M.C. , Henk, D.A. , Briggs, C.J. , Brownstein, J.S. , Madoff, L.C. , McCraw, S.L. and Gurr, S.J. (2012) Emerging fungal threats to animal, plant and ecosystem health. Nature, 484, 186–194. 22498624 10.1038/nature10947 PMC3821985

[pbi12829-bib-0015] Fuller, K.K. and Rhodes, J.C. (2012) Protein kinase A and fungal virulence. Virulence, 3, 109–121. 22460637 10.4161/viru.19396 PMC3396691

[pbi12829-bib-0016] Ghag, S.B. , Shekhawat, U.K.S. and Ganapathi, T.R. (2014) Host‐induced post‐transcriptional hairpin RNA‐mediated gene silencing of vital fungal genes confers efficient resistance against *Fusarium* wilt in banana. Plant Biotechnol. J. 12, 541–553. 24476152 10.1111/pbi.12158

[pbi12829-bib-0017] Govindarajulu, M. , Epstein, L. , Wroblewski, T. and Michelmore, R.W. (2015) Host‐induced gene silencing inhibits the biotrophic pathogen causing downy mildew of lettuce. Plant Biotechnol. J. 13, 875–883. 25487781 10.1111/pbi.12307

[pbi12829-bib-0018] Guo, J. , Dai, X. , Xu, J. , Wang, Y. , Bai, P. , Liu, F. , Duan, Y. *et al* (2011) Molecular characterization of a Fus3/Kss1 type MAPK from *Puccinia striiformis* f. sp. *tritici*, PsMAPK1. PLoS ONE, 6, e21895. 21779350 10.1371/journal.pone.0021895 PMC3136484

[pbi12829-bib-0019] Guo, J. , Bai, P. , Yang, Q. , Liu, F. , Wang, X. , Huang, L. and Kang, Z. (2013) Wheat zinc finger protein TaLSD1, a negative regulator of programmed cell death, is involved in wheat resistance against stripe rust fungus. Plant Physiol. Biochem. 71, 164–172. 23933226 10.1016/j.plaphy.2013.07.009

[pbi12829-bib-0020] Hannon, G.J. (2002) RNA interference. Nature, 418, 244–251. 12110901 10.1038/418244a

[pbi12829-bib-0021] Hartmann, H.A. , Krüger, J. , Lottspeich, F. and Kahmann, R. (1999) Environmental signals controlling sexual development of the corn smut fungus *Ustilago maydis* through the transcriptional regulator Prf1. Plant Cell, 11, 1293–1305. 10402430 10.1105/tpc.11.7.1293 PMC144278

[pbi12829-bib-0022] Hassebrauk, K. and Schroeder, J. (1964) Studies on the germination of yellow rust urediospores In Proceedings of the First European and Mediterranean Cereal Rusts Conference (Macer R. C. F. and Wolfe M. S., eds.), pp. 12–18. Cambridge UK: Plant Breeding Institute.

[pbi12829-bib-0023] Hellens, R.P. , Allan, A.C. , Friel, E.N. , Bolitho, K. , Grafton, K. , Templeton, M.D. , Karunairetnam, S. *et al* (2005) Transient expression vectors for functional genomics, quantification of promoter activity and RNA silencing in plants. Plant Methods, 1, 13. 16359558 10.1186/1746-4811-1-13 PMC1334188

[pbi12829-bib-0024] Hormaza, J.I. (2002) Molecular characterization and similarity relationships among apricot (*Prunus armeniaca* L.) genotypes using simple sequence repeats. Theor. Appl. Genet. 104, 321–328. 12582704 10.1007/s001220100684

[pbi12829-bib-0025] Hu, S. , Zhou, X. , Gu, X. , Cao, S. , Wang, C. and Xu, J. (2014) The cAMP‐PKA pathway regulates growth, sexual and asexual differentiation, and pathogenesis in *Fusarium graminearum* . Mol. Plant Microbe Interact. 27, 557–566. 24450772 10.1094/MPMI-10-13-0306-R

[pbi12829-bib-0026] Huang, Q. , Li, X. , Chen, W.Q. , Xiang, Z.P. , Zhong, S.F. , Chang, Z.J. , Zhang, M. *et al* (2014) Genetic mapping of a putative *Thinopyrum intermedium*‐derived stripe rust resistance gene on wheat chromosome 1B. Theor. Appl. Genet. 127, 843–853. 24487977 10.1007/s00122-014-2261-7

[pbi12829-bib-0027] Huvenne, H. and Smagghe, G. (2010) Mechanisms of dsRNA uptake in insects and potential of RNAi for pest control: a review. J. Insect Physiol. 56, 227–235. 19837076 10.1016/j.jinsphys.2009.10.004

[pbi12829-bib-0028] Jackson, A.L. , Bartz, S.R. , Schelter, J. , Kobayashi, S.V. , Burchard, J. , Mao, M. , Li, B. *et al* (2003) Expression profiling reveals off‐target gene regulation by RNAi. Nat. Biotechnol. 21, 635–637. 12754523 10.1038/nbt831

[pbi12829-bib-0029] Johnson, R. (1988) Durable resistance to yellow (stripe) rust in wheat and its implications in plant breeding In Breeding Strategies for Resistance to the Rusts of Wheat (Simmonds N. W. and Rajaram S., eds.), pp. 63–75. Mexico D.F.: CIMMYT.

[pbi12829-bib-0030] Kang, Z. , Huang, L. and Buchenauer, H. (2002) Ultrastructural changes and localization of lignin and callose in compatible and incompatible interactions between wheat and *Puccinia striiformis* . J. Plant Dis. Prot. 109, 25–37.

[pbi12829-bib-0031] Kang, Z.S. , Wang, Y. , Huang, L.L. , Wei, G.R. and Zhao, J. (2003) Histology and ultrastructure of incompatible combination between *Puccinia striiformis* and wheat cultivars with resistance of low reaction type. Sci. Agric. Sin. 36, 1026–1031.

[pbi12829-bib-0032] Lee, W. , Hammond‐Kosack, K.E. and Kanyuka, K. (2012) Barley stripe mosaic virus‐mediated tools for investigating gene function in cereal plants and their pathogens: virus‐induced gene silencing, host‐mediated gene silencing, and virus‐mediated overexpression of heterologous protein. Plant Physiol. 160, 582–590. 22885938 10.1104/pp.112.203489 PMC3461540

[pbi12829-bib-0033] Line, R.F. and Qayoum, A. (1992) Virulence, aggressiveness, evolution and distribution of races of Puccinia striiformis (the cause of stripe rust of wheat) in North America, pp. 1968–1987. Technical Bulletin (USA).

[pbi12829-bib-0034] Liu, F. , Guo, J. , Bai, P. , Duan, Y. , Wang, X. , Cheng, Y. , Feng, H. *et al* (2012) Wheat TaRab7 GTPase is part of the signaling pathway in responses to stripe rust and abiotic stimuli. PLoS ONE, 7, e37146. 22629358 10.1371/journal.pone.0037146 PMC3358313

[pbi12829-bib-0035] Livak, K.J. and Schmittgen, T.D. (2001) Analysis of relative gene expression data using real‐time quantitative PCR and the 2^−ΔΔCT^ method. Methods, 25, 402–408. 11846609 10.1006/meth.2001.1262

[pbi12829-bib-0036] Ma, J. , Huang, X. , Wang, X. , Chen, X. , Qu, Z. , Huang, L. and Kang, Z. (2009) Identification of expressed genes during compatible interaction between stripe rust (*Puccinia striiformis*) and wheat using a cDNA library. BMC Genom. 10, 586. 10.1186/1471-2164-10-586 PMC3087560 19995415

[pbi12829-bib-0037] Miller, S.C. , Miyata, K. , Brown, S.J. and Tomoyasu, Y. (2012) Dissecting systemic RNA interference in the red flour beetle *Tribolium castaneum*: parameters affecting the efficiency of RNAi. PLoS ONE, 7, e47431. 23133513 10.1371/journal.pone.0047431 PMC3484993

[pbi12829-bib-0038] Mitchell, T.K. and Dean, R.A. (1995) The cAMP‐dependent protein kinase catalytic subunit is required for appressorium formation and pathogenesis by the rice blast pathogen *Magnaporthe grisea* . Plant Cell, 7, 1869–1878. 8535140 10.1105/tpc.7.11.1869 PMC161045

[pbi12829-bib-0039] Nowara, D. , Gay, A. , Lacomme, C. , Shaw, J. , Ridout, C. , Douchkov, D. , Hensel, G. *et al* (2010) HIGS: host‐induced gene silencing in the obligate biotrophic fungal pathogen *Blumeria graminis* . Plant Cell, 22, 3130–3141. 20884801 10.1105/tpc.110.077040 PMC2965548

[pbi12829-bib-0040] Pan, X. and Heitman, J. (1999) Cyclic AMP‐dependent protein kinase regulates pseudohyphal differentiation in *Saccharomyces cerevisiae* . Mol. Cell. Biol. 19, 4874–4887. 10373537 10.1128/mcb.19.7.4874 PMC84286

[pbi12829-bib-0041] Panwar, V. , McCallum, B. and Bakkeren, G. (2013a) Endogenous silencing of *Puccinia triticina* pathogenicity genes through in *planta*‐expressed sequences leads to the suppression of rust diseases on wheat. Plant J. 73, 521–532. 23110316 10.1111/tpj.12047

[pbi12829-bib-0042] Panwar, V. , McCallum, B. and Bakkeren, G. (2013b) Host‐induced gene silencing of wheat leaf rust fungus *Puccinia triticina* pathogenicity genes mediated by the Barley stripe mosaic virus. Plant Mol. Biol. 81, 595–608. 23417582 10.1007/s11103-013-0022-7

[pbi12829-bib-0043] Park, G. , Xue, C. , Zhao, X. , Kim, Y. , Orbach, M. and Xu, J. (2006) Multiple upstream signals converge on the adaptor protein Mst50 in *Magnaporthe grisea* . Plant Cell, 18, 2822–2835. 17056708 10.1105/tpc.105.038422 PMC1626611

[pbi12829-bib-0044] Ren, Y. , Li, S. , Xia, X. , Zhou, Q. , He, Y. , Wei, Y. , Zheng, Y. *et al* (2015) Molecular mapping of a recessive stripe rust resistance gene *yrMY37* in Chinese wheat cultivar Mianmai 37. Mol. Breeding, 35, 1–9.

[pbi12829-bib-0045] Robertson, L.S. and Fink, G.R. (1998) The three yeast A kinases have specific signaling functions in pseudohyphal growth. Proc. Natl Acad. Sci. USA, 95, 13783–13787. 9811878 10.1073/pnas.95.23.13783 PMC24897

[pbi12829-bib-0046] Sambrook, J. and Russell, D.W. (1989) Molecular Cloning. A Laboratory Manual. Cold Spring Harbor, NY: Cold Spring Harbor Laboratory Press.

[pbi12829-bib-0047] Scofield, S.R. , Huang, L. , Brandt, A.S. and Gill, B.S. (2005) Development of a virus‐induced gene‐silencing system for hexaploid wheat and its use in functional analysis of the *Lr21*‐mediated leaf rust resistance pathway. Plant Physiol. 138, 2165–2173. 16024691 10.1104/pp.105.061861 PMC1183404

[pbi12829-bib-0048] de Souza, N. (2014) Genetics: more specific CRISPR editing. Nat. Methods, 11, 712. 25110782 10.1038/nmeth.3020

[pbi12829-bib-0049] Tamura, K. , Stecher, G. , Peterson, D. , Filipski, A. and Kumar, S. (2013) MEGA6: molecular evolutionary genetics analysis version 6.0. Mol. Biol. Evol. 30, 2725–2729. 24132122 10.1093/molbev/mst197 PMC3840312

[pbi12829-bib-0050] Thevelein, J.M. and De Winde, J.H. (1999) Novel sensing mechanisms and targets for the cAMP‐protein kinase A pathway in the yeast *Saccharomyces cerevisiae* . Mol. Microbiol. 33, 904–918. 10476026 10.1046/j.1365-2958.1999.01538.x

[pbi12829-bib-0051] Toda, T. , Cameron, S. , Sass, P. , Zoller, M. and Wigler, M. (1987) Three different genes in *S. cerevisiae* encode the catalytic subunits of the cAMP‐dependent protein kinase. Cell, 50, 277–287. 3036373 10.1016/0092-8674(87)90223-6

[pbi12829-bib-0052] Vaucheret, H. and Fagard, M. (2001) Transcriptional gene silencing in plants: targets, inducers and regulators. Trends Genet. 17, 29–35. 11163919 10.1016/s0168-9525(00)02166-1

[pbi12829-bib-0053] Voegele, R.T. and Mendgen, K. (2003) Rust haustoria: nutrient uptake and beyond. New Phytol. 159, 93–100. 10.1046/j.1469-8137.2003.00761.x 33873671

[pbi12829-bib-0054] Wang, C. , Huang, L. , Buchenauer, H. , Han, Q. , Zhang, H. and Kang, Z. (2007) Histochemical studies on the accumulation of reactive oxygen species (O^2−^ and H_2_O_2_) in the incompatible and compatible interaction of wheat‐*Puccinia striiformis* f. sp. *tritici* . Physiol. Mol. Plant Pathol. 71, 230–239.

[pbi12829-bib-0055] Xu, J. , Urban, M. , Sweigard, J.A. and Hamer, J.E. (1997) The *CPKA* gene of *Magnaporthe grisea* is essential for appressorial penetration. Mol. Plant Microbe Interact. 10, 187–194.

[pbi12829-bib-0056] Xu, J. , Linning, R. , Fellers, J. , Dickinson, M. , Zhu, W. , Antonov, I. , Joly, D.L. *et al* (2011) Gene discovery in EST sequences from the wheat leaf rust fungus *Puccinia triticina* sexual spores, asexual spores and haustoria, compared to other rust and corn smut fungi. BMC Genom. 12, 1. 10.1186/1471-2164-12-161 PMC3074555 21435244

[pbi12829-bib-0057] Yin, C. , Chen, X. , Wang, X. , Han, Q. , Kang, Z. and Hulbert, S.H. (2009) Generation and analysis of expression sequence tags from haustoria of the wheat stripe rust fungus *Puccinia striiformis* f. sp. *tritici* . BMC Genom. 10, 1. 10.1186/1471-2164-10-626 PMC2805700 20028560

[pbi12829-bib-0058] Yin, C. , Jurgenson, J.E. and Hulbert, S.H. (2011) Development of a host‐induced RNAi system in the wheat stripe rust fungus *Puccinia striiformis* f. sp. *tritici* . Mol. Plant Microbe Interact. 24, 554–561. 21190437 10.1094/MPMI-10-10-0229

[pbi12829-bib-0059] Zarnack, K. , Eichhorn, H. , Kahmann, R. and Feldbrügge, M. (2008) Pheromone‐regulated target genes respond differentially to MAPK phosphorylation of transcription factor Prf1. Mol. Microbiol. 69, 1041–1053. 18627457 10.1111/j.1365-2958.2008.06345.x

[pbi12829-bib-0060] Zheng, X. , Zhu, J. , Kapoor, A. and Zhu, J.K. (2007) Role of Arabidopsis AGO6 in siRNA accumulation, DNA methylation and transcriptional gene silencing. EMBO J. 26, 1691–1701. 17332757 10.1038/sj.emboj.7601603 PMC1829372

[pbi12829-bib-0061] Zheng, W.M. , Huang, L.L. , Huang, J.Q. , Wang, X.J. , Chen, X.M. , Zhao, J. , Guo, J. *et al* (2013) High genome heterozygosity and endemic genetic recombination in the wheat stripe rust fungus. Nat. Commun. 4, 2673. 24150273 10.1038/ncomms3673 PMC3826619

