# Supplementary figures and images for: Host‐induced gene silencing of an important pathogenicity factor PsCPK1 in Puccinia striiformis f. sp. tritici enhances resistance of wheat to stripe rust
Source: Plant Biotechnol J. 2017 Oct 23;16(3):797–807. doi: 10.1111/pbi.12829 (PMC5814584; doi:10.1111/pbi.12829)

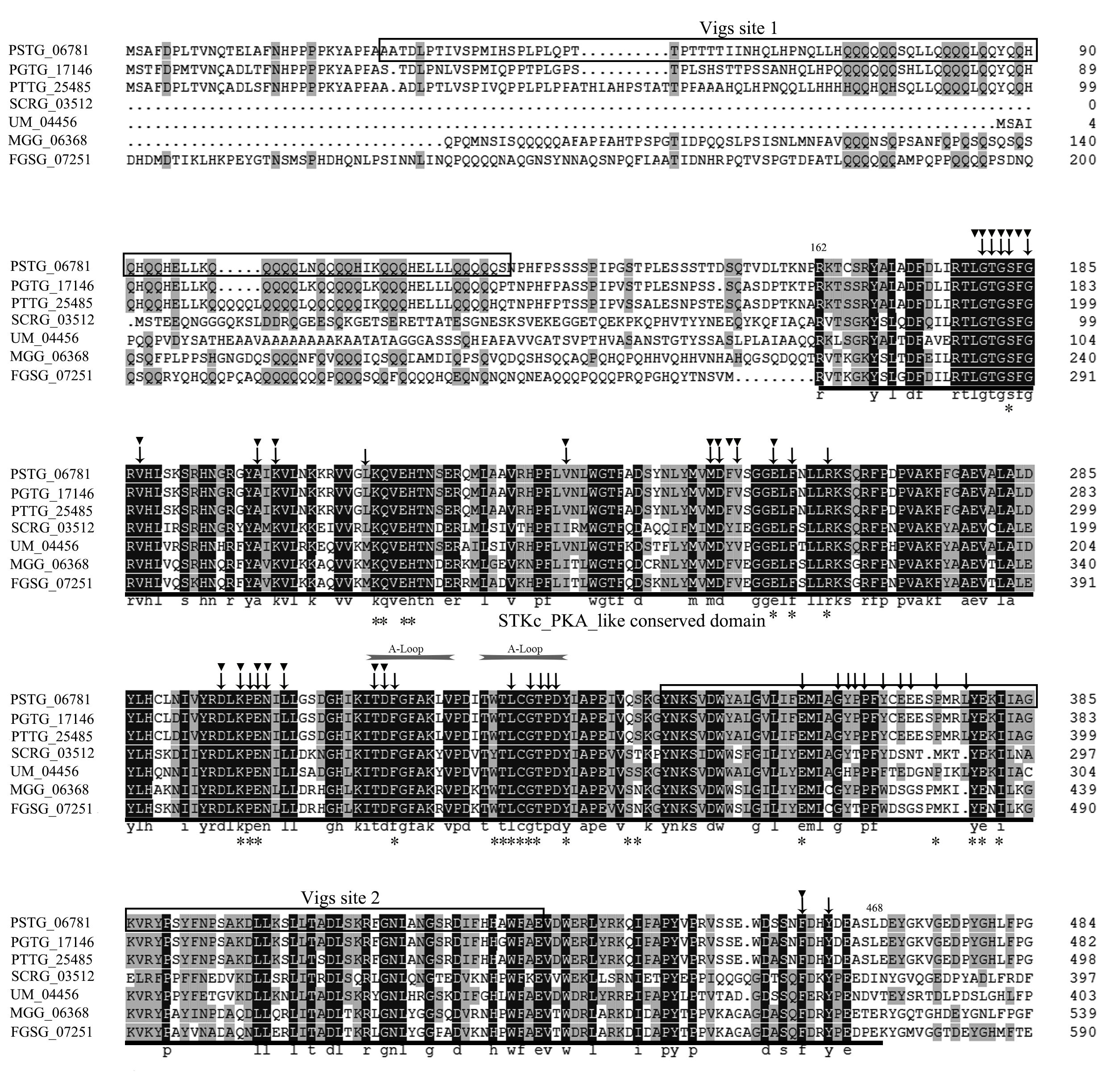

Supplement: Supplementary file 1 — Figure S1 Amino acid sequence alignments of PsCPK1 with other fungal catalytic subunits of PKA. [file PBI-16-797-s006.tif]

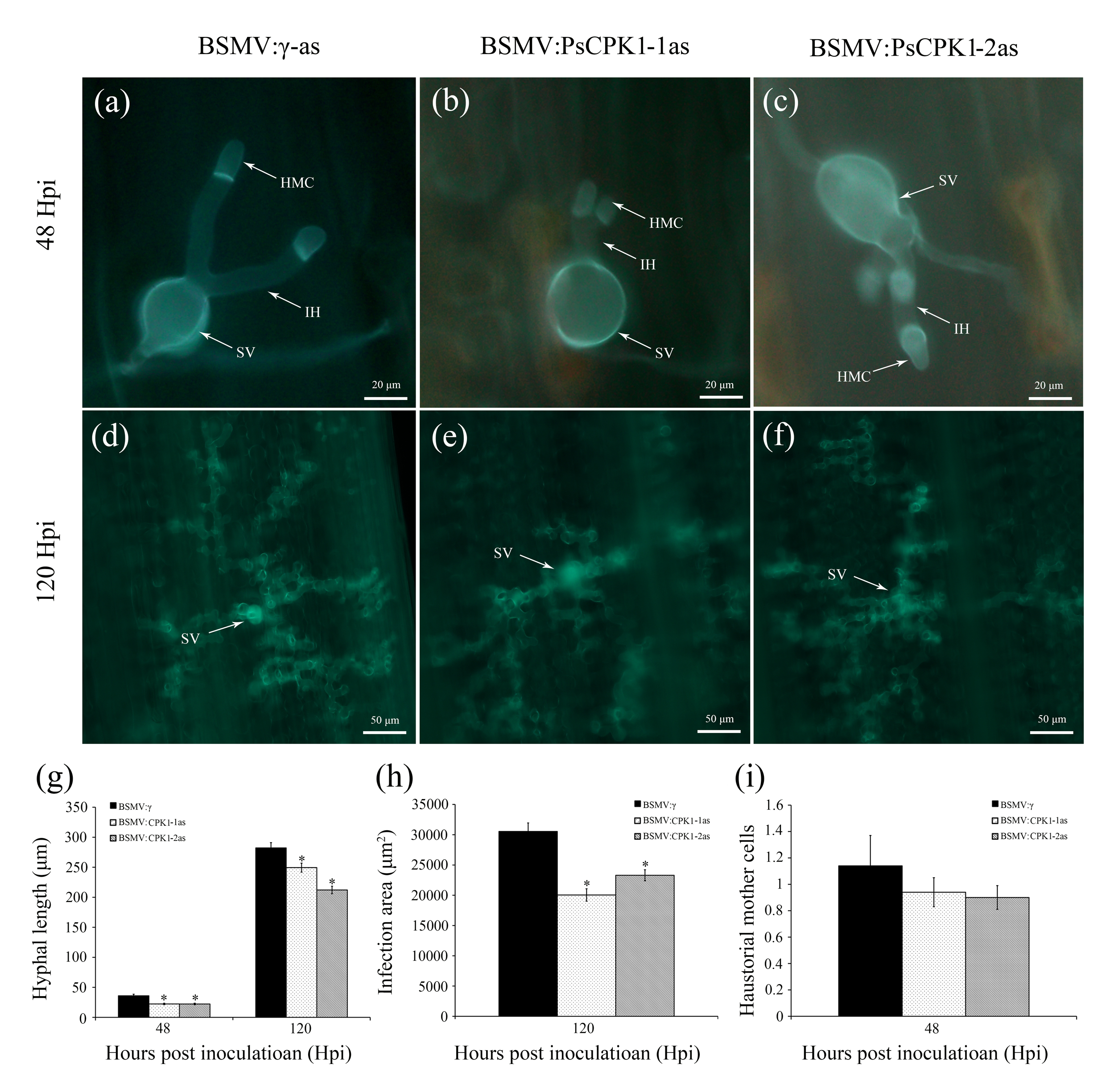

Supplement: Supplementary file 2 — Figure S2 Histological observation of fungal growth in PsCPK1‐knockdown wheat plants after inoculation with Pst isolate CYR32. [file PBI-16-797-s010.tif]

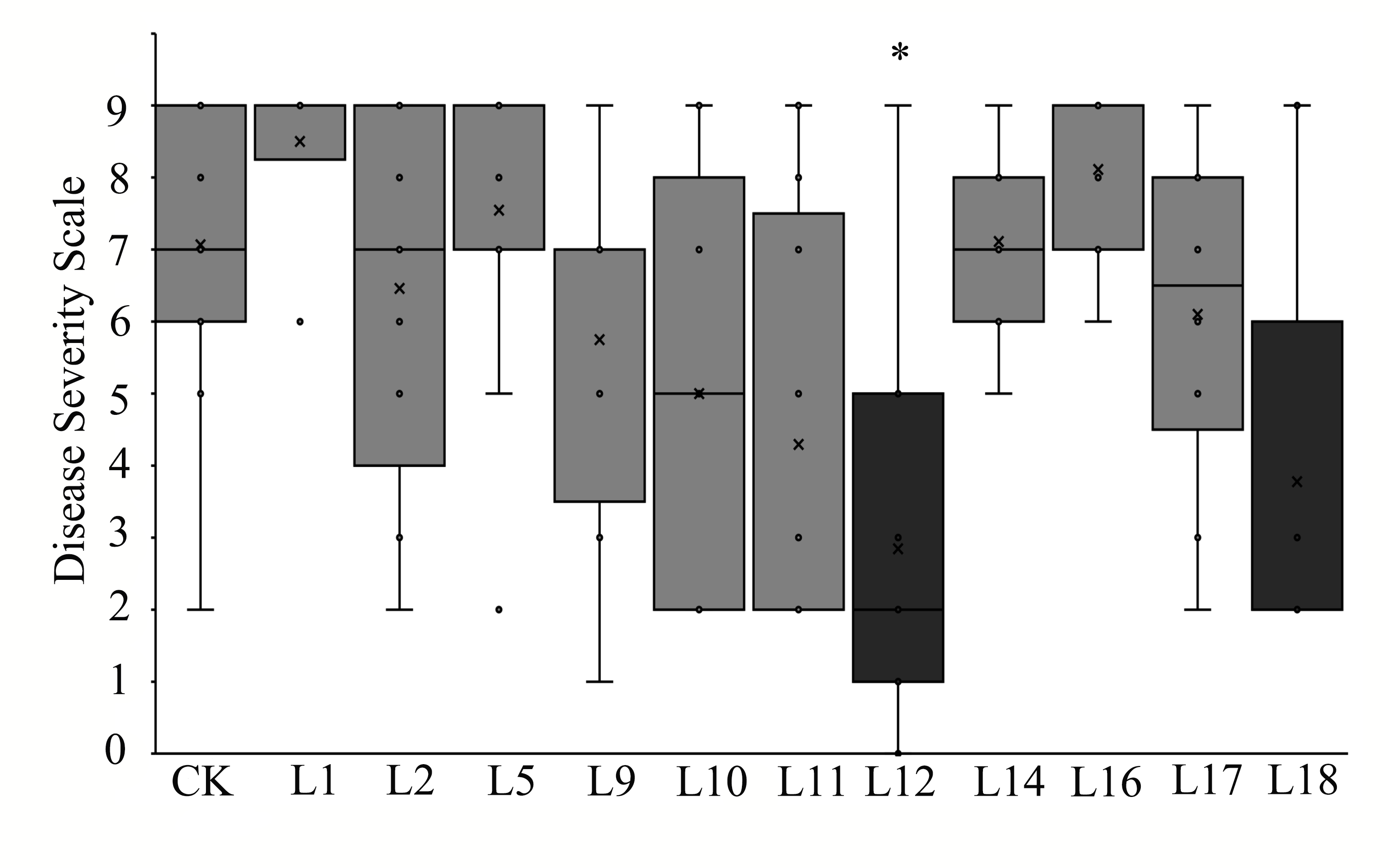

Supplement: Supplementary file 3 — Figure S3 Bioassay of the transgenic wheat plants for Pst resistance. [file PBI-16-797-s009.tif]

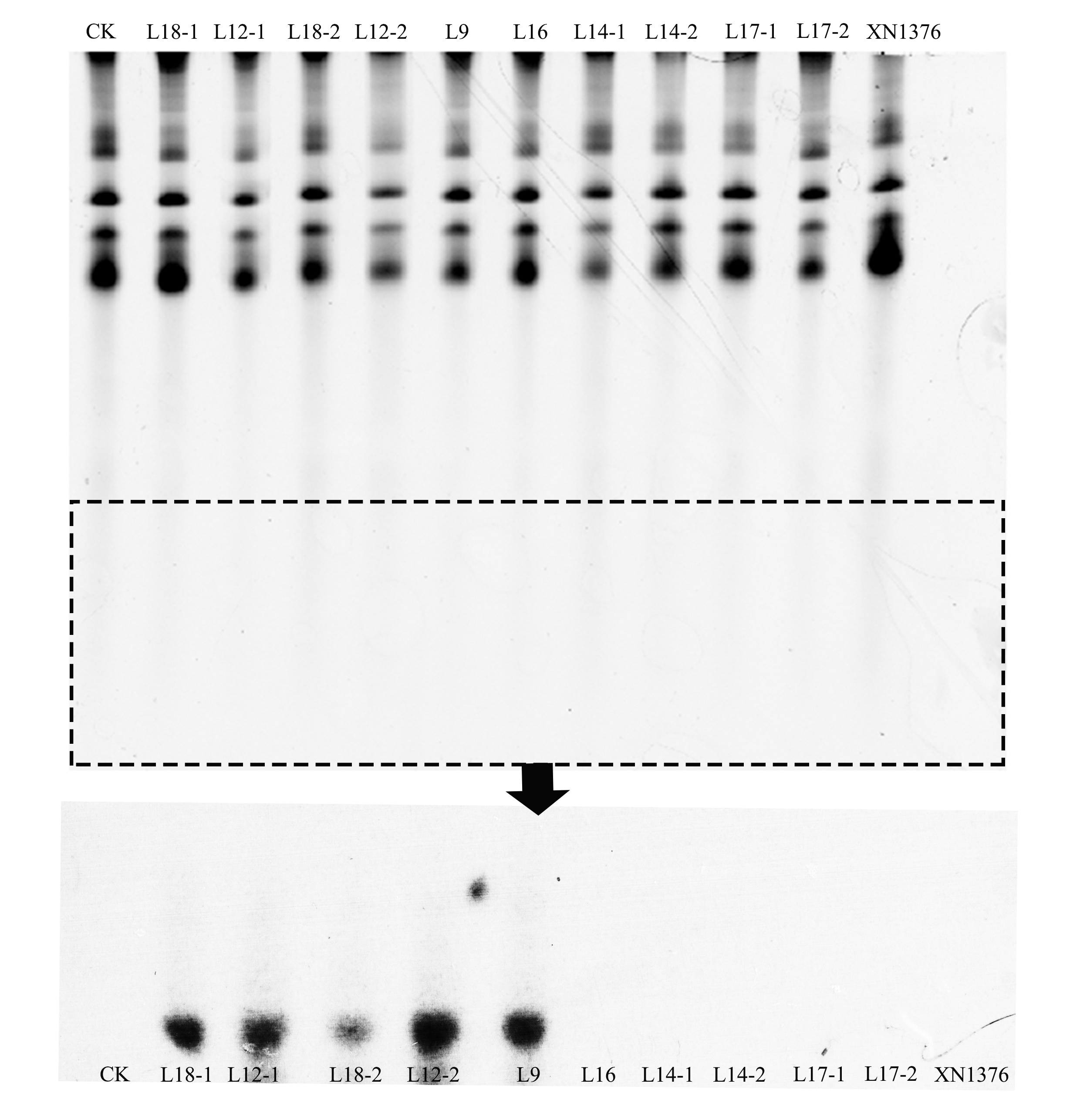

Supplement: Supplementary file 4 — Figure S4 The expression of the small RNA in T4 generation lines was analysed by RNA gel blotting. [file PBI-16-797-s011.tif]

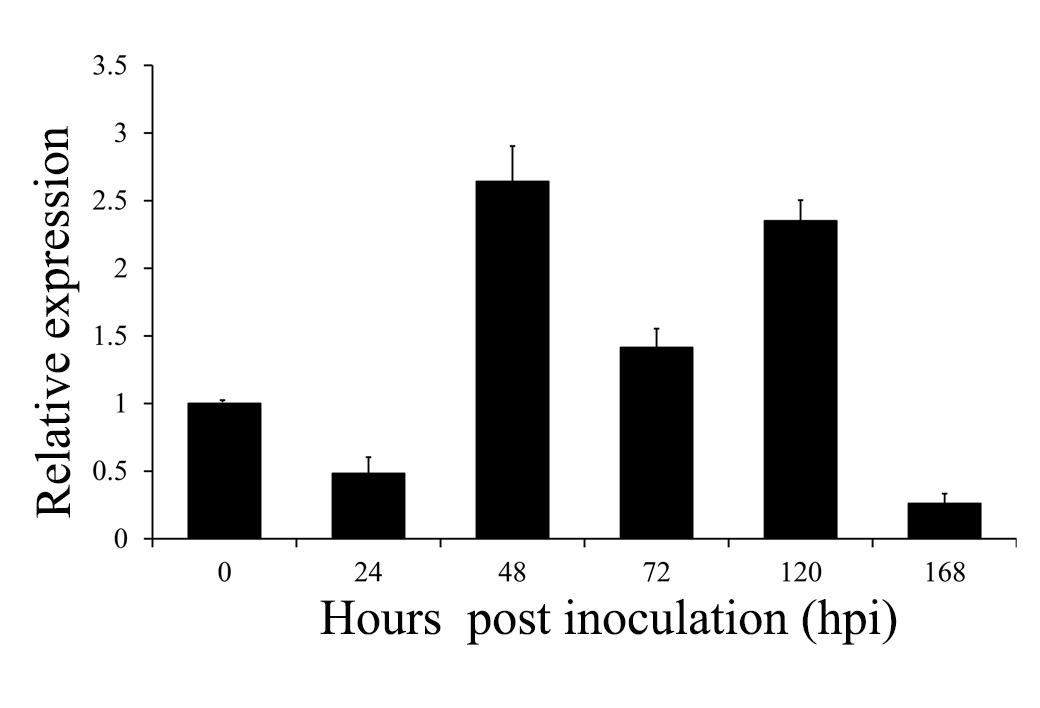

Supplement: Supplementary file 5 — Figure S5 Transcript profiles of PsCPK1 in the leaves of wheat cultivar XN1376 infected by Pst isolate CYR32. [file PBI-16-797-s001.tif]

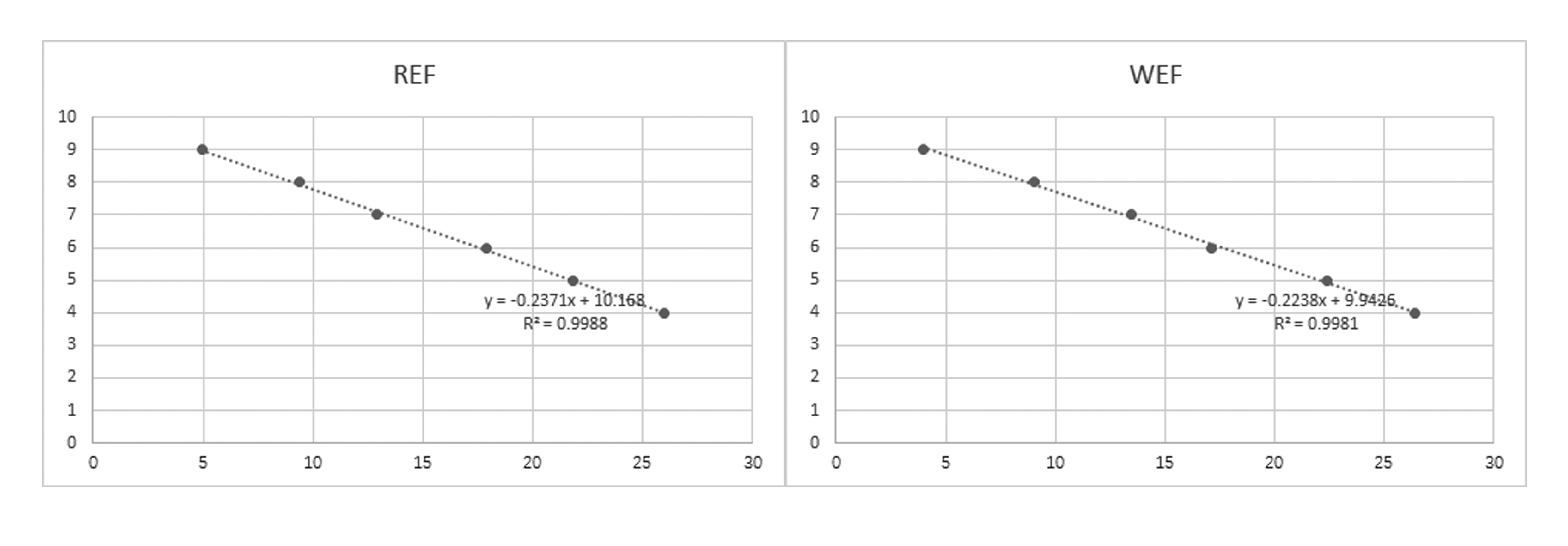

Supplement: Supplementary file 6 — Figure S6 Standard curves generated for the absolute quantification of Pst (A) and wheat (B). [file PBI-16-797-s002.tif]

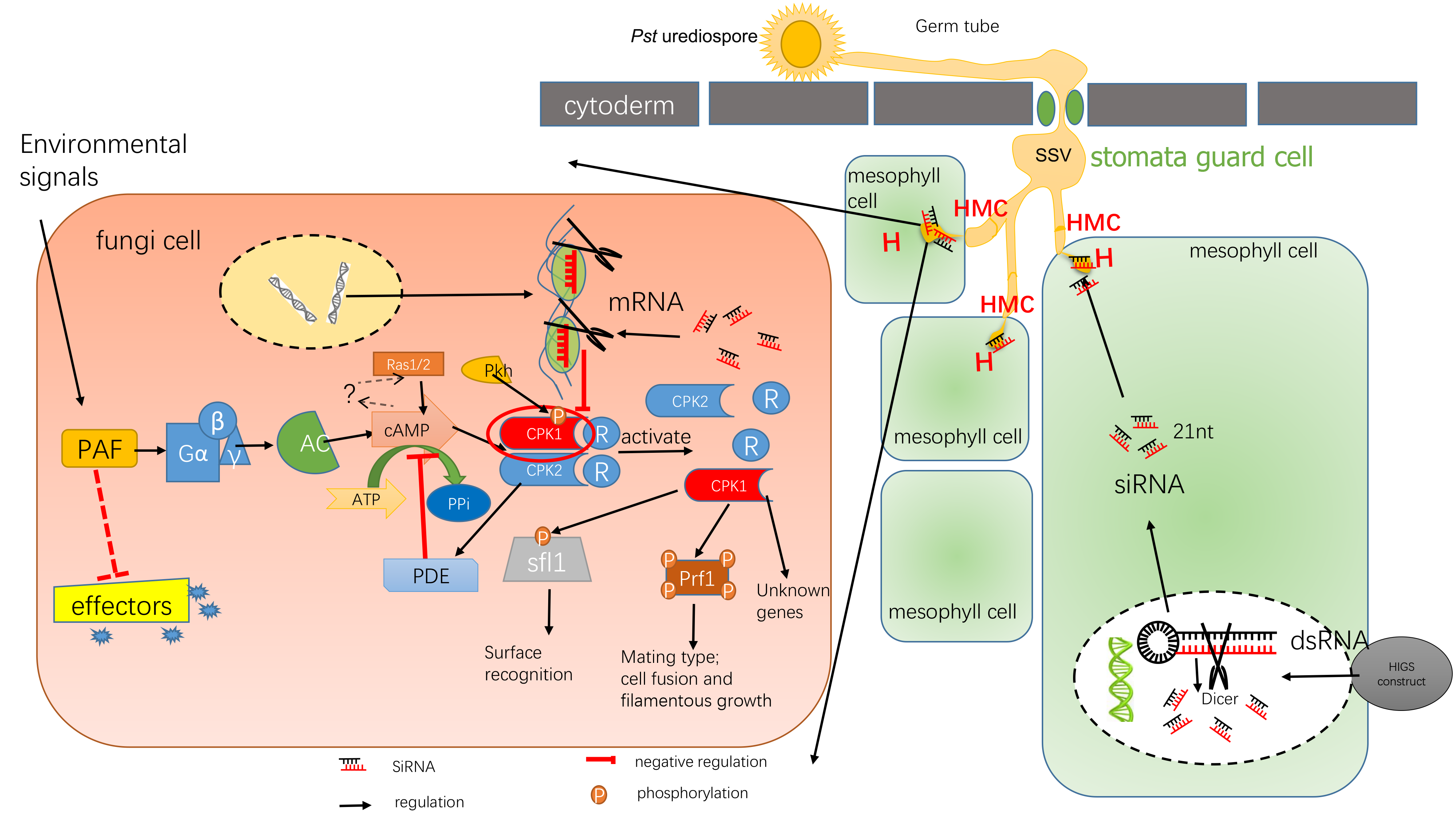

Supplement: Supplementary file 7 — Figure S7 Schematic presentation of possible HIGS mechanisms involved in PKA pathway. [file PBI-16-797-s003.tif]

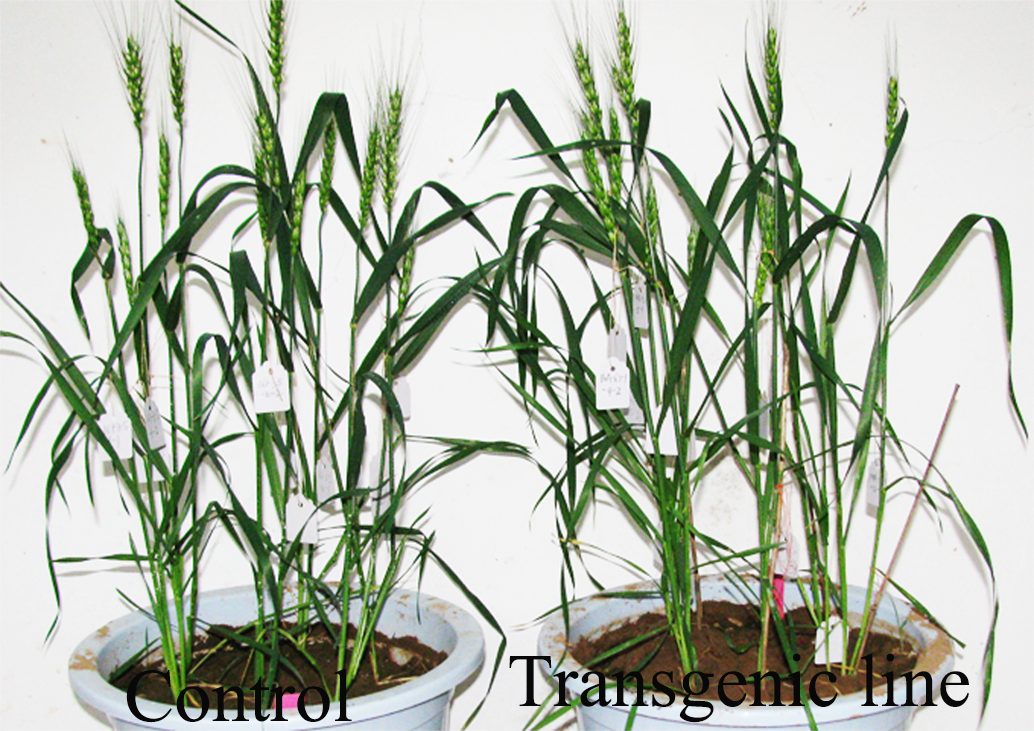

Supplement: Supplementary file 8 — Figure S8 No significant difference was observed for the growth between transgenic and control wheat lines. [file PBI-16-797-s004.tif]
